# The health sector and fiscal policies of fossil fuels: an essential alignment for the health and climate change agenda

**DOI:** 10.1136/bmjgh-2023-012938

**Published:** 2023-10-09

**Authors:** Douglas Webb, Odd N Hanssen, Robert Marten

**Affiliations:** 1 HIV and Health Group, United Nations Development Programme, Amman, Jordan; 2 Sustainable Financing Hub, United Nations Development Programme, New York, New York, USA; 3 WHO Alliance for Health Policy and Systems Research, Geneva, Switzerland

**Keywords:** Environmental health, Health economics, Health policy, Public Health

**Summary Box**

- The relationship between climate change and adverse health impacts is driven principally the combustion of fossil fuels. Yet the health sector’s engagement with fossil fuel policy has largely been restricted to health facility decarbonisation. Health sector action can focus on three pillars;
- Framing fossil fuel fiscal policy as a dominant commercial determinant of health;
- Advocating for and co-creating systemic legislative reform; and
- Direct support to fossil fuel subsidy reform (FFSR).
- While increasing engagement in reform, the health sector must ensure that health inequities are not increased through the appropriate design of social protection mechanisms and protection guarantees for the health sector itself, especially in its service delivery capacity to most vulnerable populations.

The WHO declared in 2018 that ‘climate change is the greatest challenge of the 21st century, threatening human health and development’.^1^ The relationship between climate change and adverse health impacts has been described in detail^2^ and is driven by increasing greenhouse gas emissions (especially CO_2_, black carbon, methane and nitrous oxides) principally from the combustion of fossil fuels.^3^ The relationship between climate change and health has focused on the three notions of (1) mitigation, or reducing the climate footprint of the health sector itself, (2) health sector adaptation or improving the resilience of the health sector in the face of climate-related hazards and (3) the multitude of direct and indirect impacts of climate change on the global disease burden. For example, a conservative figure is 250 000 additional deaths each year due to climate change projected for between 2030 and 2050.^4^ This number is surely an underestimation, given the continuing climatic effects with devastating effects on human life, such as the 2022 floods in Pakistan. Closely connected are the economic and health costs of air pollution from burning fossil fuels, which totalled an estimated US$2.9 trillion in 2018, when 7 million deaths, work absences, years of life lost and premature deaths are considered together. This cost represents a minimum of 3.3% of global GDP, or about US$8 billion per day.^5^

The inter-relationship between ill-health, health inequities and fossil fuels is arguably today’s single most important social determinant of health. Continued unabated fossil fuel consumption and accompanying fiscal policies should be of singular concern, especially to the health sector. Yet the health sector’s engagement with fossil fuel policy and action has, however, been largely limited to health sector decarbonisation. While this is itself an important contribution to climate change mitigation, the health sector is the source of roughly 5.2% of greenhouse gas (GHG) emissions (estimated to be much higher in some countries—7% in Australia and 10% in the USA)^6–8^ compared with 73% from the energy sector.^9^ The health sector itself must urgently pursue a vigorous and unabashed advocacy and integrationist agenda, driven by its own growing legitimacy as a driver of meaningful decarbonisation practice.^10^ Redressing fossil fuel-supportive fiscal policies should be the priority of these efforts.

The fossil fuel industry benefits from subsidies of US$11 million every minute, or US$5.9 trillion (6.8% of GDP) in 2020.^11^ Instead of taxing the cost of emissions, these subsidies represent an extraordinary market failure. Rectifying this should be the focus of the health sector’s efforts and a central part of the global ‘health taxes’ agenda. While the framing of climate change as a form of ‘structural violence’ is not widely accepted, the label may be more applicable to fossil fuel subsidies. The deliberation and action of subsidisation in its various forms results from both the dominance of industrial and corporate power and the active subversion of the climate-justice complex. The power asymmetry, in the context of record fossil fuel company profits (eg, net profits earned by the five majors—Shell, Chevron, ExxonMobil, BP and TotalEnergies—surpassed US$150 billion in 2022),^12^ driven by the war in Ukraine and the subsequent escalation of prices, is widening.^13^

While at the COP26 in Glasgow, governments committed to phase down the use of ‘unabated’ coal power and to phase out ‘inefficient” fossil fuel subsidies, the health-specific commitments made^14^ relate to the vital but still limited responsibilities of the health sector. Within nationally determined contributions (NDCs) the specification of health targeted actions is, on analysis, limited at best.^15^ Despite health advocates’ voice and representation rising in recent years in the United Nations Framework Convention on Climate Change (UNFCCC) process, the overarching COP governance structure leaves little space for health accountability, monitoring and obligation. Indeed, the current triangular framing of ‘mitigation’, ‘adaptation’ and the newly endorsed momentum towards rectifying ‘loss and damage’ does not recognise or prioritise the *cobenefits* of moving from fossil fuel subsidy to fossil fuel taxation.^16^ Cobenefits entail the notion of offset; improved air quality alone could realise health benefits that easily offset the global costs of emissions reductions.^17^ This would also include reduced consumption, with its associated health benefits. Reduced subsidies would mean increased fiscal space for social spending on the Sustainable Development Goals.

Fossil fuels are a litmus test for whole-of-society approaches that are compelled to assess how to reduce, mitigate and manage health-harming processes and activities inherent within society, tackle adverse externalities and not view them as inevitable by-products of development. Our crossing of planetary boundaries and witnessing of the rise of non-communicable diseases and climate change-induced health impacts mean that pollution, carbon emissions and health harming products and processes are not being adequately managed at societal scale. Bearing their mounting costs is a deliberate choice. They are not technical failures but rather governance ones, including the avoidance of confronting the adverse commercial determinants of health.

## Policy and action agenda

Increased advocacy and action of the health sector can be built on a pre-existing platform of trust that public health professionals and institutions enjoy^18^ and must go well beyond the confines of the UNFCCC COP deliberations. A priority avenue for action is recognising that fossil fuel fiscal policy represents perhaps the single largest *commercial* determinant of present and future health. The *BMJ* and fossil fuel divestment initiative is a valuable move in this regard.^19^ Precedents are available. For example, the WHO Framework Convention on Tobacco Control limits engagement with the tobacco industry (Article 5.3), aided by surveillance tools such as the Global Tobacco Industry Interference Index.^20^ The normative and systemic protection of policy-making from interference by industry actors with clear vested interests is in its infancy, judging by the perpetuation of subsidies themselves as well as the overbearing presence of a reported 636 industry representatives in the COP27 deliberations.^21^ The health sector and its civil society allies would be well placed to initiate witnessing processes and interference indices that enable a better systemic understanding of the nature and extent of fossil fuel industry interference in the ecosystem of policy, legislative and financing process directly connected, but not limited to, UNFCCC instruments.

A second route of engagement is in the legal domain, following the July 2022 United Nations General Assembly recognition that a clean, healthy and sustainable environment is a human right. While the right to a healthy environment remains undefined in legislative terms,

the right is generally understood to include substantive and procedural elements. The substantive elements include clean air; a safe and stable climate; access to safe water and adequate sanitation; healthy and sustainably produced food; non-toxic environments in which to live, work, study and play; and healthy biodiversity and ecosystems.^22^

The development of framework legislation instruments, most critically at the national level, should be driven by coherent advocacy by the health sector, while it also advances its own environmental protection agenda. In a structured transition, forms of subsidisation should ultimately be reduced and eliminated and become replaced with taxation.

Third, direct support to the fossil fuel subsidy reform (FFSR) agenda at multiple levels is required. This would highlight, above all, the health equity dimensions, which supplement and reiterate a climate justice frame. But further action is needed, as the world needs to move beyond addressing fossil fuel subsidies towards imposing fossil fuel taxes, as part of a larger carbon pricing policy arena, towards the overall aim of the correct pricing of carbon.

Additional taxes on the point of sale of fossil fuels are also needed. These would internalise the external costs of their consumption. While the vast majority of countries that are fossil-fuel producers have subsidies on their sale, importantly most *do* levy taxes on fossil fuel consumption. These, however, are wholly insufficient. For example, the Organisation for Economic Cooperation and Development (OECD) found that 97% of energy-related CO_2_ emissions in 2019 (outside of road transport) are taxed far below levels that would reflect their true cost, even if only considering the cost of environmental impacts.^23^

Subsidy reduction, elimination and implementation of taxation should initially target the most environmentally harmful pollutants and the fuels on which the poorest households are the least dependent.^24^ Entry points within the UNFCCC do exist; 96 of the 146 NDCs refer to carbon pricing as a ‘policy option’. Over 40 countries implemented subsidy reform as of 2020, and over 60 carbon pricing initiatives have been initiated.^25^

While engaging in complex reform, it is imperative to recognise that although the world’s poorest communities and households are disproportionately affected by the impacts of climate change, these groups are also potentially most impacted by poorly designed reforms in the absence of social protection remediation measures. Rising costs of fuel would increase energy poverty levels in the socioeconomic groups most sensitive to price changes, potentially increasing health inequities, without offsets driven by social assistance that specifically targets affected groups. Understanding the degree of sensitivity and associated health equity implications of reform is essential, as research shows that it is inconsistent and unevenly applied.^26^ The contribution of the health sector could include the calibration of the extent and distribution of pre-existing health burdens and health-related costs of emissions/pollutants by source in any one administrative context and their integration into FFSR dialogue. Arguments could also be made for the (initial) exemption of the health sector as an energy consumer so as not to undermine its ability to maintain and expand service delivery capacity or to undermine efforts towards the achievement of universal health coverage.

As the March 2023 IPC report^27^ makes clear, the solutions to climate change are known and the challenges are less technical than political. The loud and unambiguous voice of the health sector in reducing fossil fuel consumption is needed now. Leaving it to others is a failing strategy.

## Data Availability

All data relevant to the study are included in the article.

## References

[1] World Health Organization. Health and climate change. 2018. Available: https://www.who.int/news-room/facts-in-pictures/detail/health-and-climate-change

[2] Romanello M, Di Napoli C, Drummond P, et al. The 2022 report of the lancet countdown on health and climate change: health at the mercy of fossil fuels. Lancet 2022;400:1619–54. 10.1016/S0140-6736(22)01540-9 36306815 PMC7616806

[3] Solomon CG, Salas RN, Malina D, et al. Fossil-fuel pollution and climate change - A new NEJM group series. N Engl J Med 2022;386:2328–9. 10.1056/NEJMe2206300 35704486

[4] World Health Organization. Climate change. n.d. Available: https://www.who.int/health-topics/climate-change#tab=tab_1

[5] Farrow A, Miller KA, Myllyvirta L. Toxic air: the price of fossil fuels. Seoul Greenpeace Southeast Asia; Available: https://www.greenpeace.org/southeastasia/publication/3603/toxic-air-the-price-of-fossil-fuels-full-report/

[6] Romanello M, Di C, Drummond P, et al. Op cit. n.d. Available: https://www.lancetcountdown.org/

[7] Malik A, Lenzen M, McAlister S, et al. The carbon footprint of Australian health care. Lancet Planet Health 2018;2:e27–35. 10.1016/S2542-5196(17)30180-8 29615206

[8] Eckelman MJ, Sherman J. Environmental impacts of the U.S. health care system and effects on public health. PLoS One 2016;11:e0157014. 10.1371/journal.pone.0157014 27280706 PMC4900601

[9] United Nations Development Programme. The state of climate change ambition. New York, 2022. Available: https://www.undp.org/publications/state-climate-ambition

[10] Rasheed FN, Baddley J, Prabhakaran P, et al. Decarbonising healthcare in low and middle income countries: potential pathways to net zero emissions. BMJ 2021;375:n1284. 10.1136/bmj.n1284 34753746 PMC8576604

[11] Parry IWH, Black S, Vernon N. Still not getting energy prices right: a global and country update of fossil fuel subsidies. Washington DC International Monetary Fund; 2021. Available: https://www.imf.org/en/Publications/WP/Issues/2021/09/23/Still-Not-Getting-Energy-Prices-Right-A-Global-and-Country-Update-of-Fossil-Fuel-Subsidies-466004#:~:text=IMF%20Working%20Papers&text=Globally%2C%20fossil%20fuel%20subsidies%20were,percent%20of%20GDP%20in%202025

[12] Several actors, including the Secretary General of the UN have called for one-time ‘windfall’ taxes on the profits of fossil fuel companies, capturing popular sentiment on the injustice of the amassing of wealth by the sector in a period where households, in lower income groups in particular, are struggling. n.d. Available: https://www.theguardian.com/world/2022/sep/20/un-secretary-general-tax-fossil-fuel-companies-climate-crisis

[13] King B. Why are BP, shell, and other oil giants making so much money right now? BBC News; 2023. Available: https://www.bbc.com/news/business-64583982

[14] World Health Organization. Alliance for action on climate change and health (ATACH). n.d. Available: https://www.who.int/initiatives/alliance-for-transformative-action-on-climate-and-health/cop26-health-programme

[15] Dasandi N, Graham H, Lampard P, et al. Engagement with health in national climate change commitments under the Paris agreement: a global mixed-methods analysis of the nationally determined contributions. Lancet Planet Health 2021;5:e93–101. 10.1016/S2542-5196(20)30302-8 33581071 PMC7887662

[16] Allan JI. Advocating for health and climate. Bull World Health Organ 2023;101:158–60. 10.2471/BLT.22.289283 36733631 PMC9874372

[17] Romanello M, Di C, Drummond P, et al. Op CIT. Https://Www.Lancetcountdown.Org/ and global climate and health alliance. cradle to grave: the health harms of fossil fuel dependence and the case for a just phase-out. 2022. Available: https://climateandhealthalliance.org/wp-content/uploads/2022/07/Cradle-To-Grave-Fossil-Fuels-Brief.pdf

[18] Sarasohn-Kahn J. People have lost trust in healthcare systems because of COVID. How can the damage be healed? World Economic Forum; 2022. Available: https://www.weforum.org/agenda/2022/03/trust-health-economy-pandemic-covid19

[19] Abbasi K, Godlee F. Investing in humanity: the BMJ’s divestment campaign. BMJ 2020;368:m167. 10.1136/bmj.m167 31974075

[20] Assunta M. Global tobacco industry interference index 2021. Global center for good governance in tobacco control (GGTC). Bangkok, Thailand Global Center for Good Governance in Tobacco Control (GGTC); 2021. Available: https://exposetobacco.org/global-index/

[21] Taylor A. COP27 awash with fossil fuel representatives, research shows. The Washington Post, WP Company; 2022. Available: https://www.washingtonpost.com/world/2022/11/10/cop27-egypt-fossil-fuel/

[22] Office of the High Commissioner for Human Rights, United Nations Environment Programme, United Nations Development Programme. What is a right to a healthy environment? Information note. New York UNDP; 2023. Available: https://www.undp.org/publications/what-right-healthy-environment

[23] Organisation for Economic Cooperation and Development. Taxing energy use 2019: using taxes for climate action. Paris: OECD Publishing, 2019.

[24] United Nations Development Programme. A guide to carbon pricing and fossil fuel subsidy reform. New York UNDP; 2021. Available: https://www.undp.org/publications/guide-carbon-pricing-and-fossil-fuel-subsidy-reform

[25] World Bank Group. State and trends of carbon pricing. Washington DC World Bank; 2021. Available: https://openknowledge.worldbank.org/handle/10986/35620

[26] Feng K, Hubacek K, Liu Y, et al. Managing the distributional effects of energy taxes and subsidy removal in Latin America and the Caribbean. Appl Energy 2018;225:424–36. 10.1016/j.apenergy.2018.04.116

[27] Intergovernmental Panel on Climate Change. AR6 synthesis report: climate change. 2023. Available: https://www.ipcc.ch/report/sixth-assessment-report-cycle/

